# Age of acquisition effects on traditional Chinese character naming and lexical decision

**DOI:** 10.3758/s13423-020-01787-8

**Published:** 2020-08-12

**Authors:** Ya-Ning Chang, Chia-Ying Lee

**Affiliations:** 1 MRC Cognition and Brain Sciences Unit, University of Cambridge, Cambridge, CB2 7EF UK; 2 Institute of Linguistics, Academia Sinica, Taipei, Taiwan; 3 Institute of Cognitive Neuroscience, National Central University, Taoyuan, Taiwan; 4 Research Center for Mind, Brain, and Learning, National Chengchi University, Taipei, Taiwan

**Keywords:** Age of acquisition, Character naming, Lexical decision, Chinese lexical processing

## Abstract

Across languages, age of acquisition (AoA) is a critical psycholinguistic factor in lexical processing, reflecting the influence of learning experience. Early-acquired words tend to be processed more quickly and accurately than late-acquired words. Recently, an integrated view proposed that both the mappings between representations and the construction of semantic representations contribute to AoA effects, thus, predicting larger AoA effects for words with arbitrary mappings between representations as well as for tasks requiring greater semantic processing. We investigated how these predictions generalize to the Chinese language system that differs from alphabetic languages regarding the ease of mappings and semantic involvement in lexical processing. A cross-task investigation of differential psycholinguistic effects was conducted with large character naming and lexical decision datasets to establish the extent to which semantics is involved in the two tasks. We focused on examining the effect sizes of lexical-semantic variables and AoA, and the interaction between AoA and consistency. The results demonstrated that semantics influenced Chinese character naming more than lexical decision, which is in contrast with the findings related to English language, though, critically, AoA effects were more pronounced for character naming than for lexical decision. Additionally, an interaction between AoA and consistency was found in character naming. Our findings provide cross-linguistic evidence supporting the view of multiple origins of AoA effects in the language-processing system.

**Electronic supplementary material:**

The online version of this article (10.3758/s13423-020-01787-8) contains supplementary material, which is available to authorized users.

## Introduction

Linguistic experience has a profound effect on language acquisition and processing pertaining to how language is learned and used, thus affecting cognition and social interaction (Kidd, Donnelly, & Christiansen, 2018). Chronological experience of vocabulary acquisition is one such language experience. Age of acquisition (AoA) effects refer to observations that words learned early in life are processed more quickly and accurately than words learned later in life. These effects have been reported to affect multiple levels of language processing as is evident from their influence on a variety of language tasks, such as word naming, lexical decision, picture naming, semantic relatedness, and naturalistic reading (Brysbaert, Wijnendaele, & Deyne, 2000; Cortese & Khanna, 2007; Davies, Arnell, Birchenough, Grimmond, & Houlson, 2017; Dirix & Duyck, 2017; Ghyselinck, Lewis, & Brysbaert, 2004; Monaghan & Ellis, 2002). The same has also been demonstrated in electroencephalography and neuroimaging studies (Bakhtiar, Su, Lee, & Weekes, 2016; Ellis, Burani, Izura, Bromiley, & Venneri, 2006; Woollams, 2012; Yum & Law, 2019), as well as by computational modeling (Chang, Monaghan, & Welbourne, 2019; Ellis & Lambon Ralph, 2000; Monaghan & Ellis, 2010; Steyvers & Tenenbaum, 2005; Zevin & Seidenberg, 2002).

There has been criticism of the genuineness of AoA effects since they are correlated with other lexical-semantic variables (e.g., frequency and concreteness) (Strain, Patterson, & Seidenberg, 1995; Zevin & Seidenberg, 2004). However, when all the key variables are considered, AoA effects cannot be reduced to the related variables (Brysbaert, 2017; Chang et al., 2019; Cortese & Khanna, 2007; Davies et al., 2017). A recent review by Brysbaert and Ellis (2016) reported a larger AoA effect for tasks with more semantic involvement, and highlighted that a similar pattern was observable for frequency effects. As the similar patterns of psycholinguistic effects are suggestive of similarities in the functional locus in the system (Adelman, Sabatos-DeVito, Marquis, & Estes, 2014), AoA may be coupled with frequency and may have a common origin in the language-processing system.

### Theoretical accounts of AoA

Why do early-learned words enjoy a processing privilege in lexical processing? *The representation theory* argues that AoA effects could be attributed to the incremental construction of semantic representations (Brysbaert & Ghyselinck, 2006; Steyvers & Tenenbaum, 2005). Early-learned words tend to develop stronger connections with other words as they have richer semantic representations, and are thus more resistant to cognitive impairment (Brysbaert & Ellis, 2016; Ellis, 2012; Woollams, 2012). Key evidence supporting this theory is that the effect size of AoA depends on semantic involvement of the tasks, thus, AoA effects are generally larger for picture naming than for lexical decision, followed by word naming (Juhasz, 2005).

Alternatively, *the mapping theory* proposes that AoA effects could result from reduced neuroplasticity during the learning of mappings between representations over time (Ellis & Lambon Ralph, 2000; Monaghan & Ellis, 2010). Early-learned words are processed using the rich resources available in the system, whereas late-learned words need to be fitted into the system already tuned to early-learned words. Consequently, there is a processing cost for late-learned words, particularly for those having mapping structures that are different to the early-learned words (Lambon Ralph & Ehsan, 2006; Zevin & Seidenberg, 2002). Critical evidence supporting this theory is that AoA effects are stronger for words with inconsistent print-to-sound mappings (e.g., *suite*) than for words with consistent print-to-sound mappings (e.g., *swell*) in word naming (Monaghan & Ellis, 2002).

An emerging view is that AoA effects can be observed as a consequence of incremental learning, resulting from both the construction of representations and changing plasticity in the learning system (Brysbaert & Ellis, 2016; Chang et al., 2019; Dirix & Duyck, 2017; Menenti & Burani, 2007). Using a computational model of reading across development, Chang et al. (2019) demonstrated that the key evidence for the representation theory (stronger AoA effects for lexical decision than for word naming) and the mapping theory (interaction between AoA and consistency in word naming) of AoA could be observed simultaneously during incremental learning. The results provide support for the integrated view of AoA, suggesting that lexical processing is gradually shaped by experience of learning during development as a consequence of more connections and accessibility of early- than of late-learned words (Brysbaert & Ellis, 2016). If, as argued by the integrated view of AoA, the experience of learning has an impact on both representations and mappings in lexical processing, the argument can be further strengthened by an assessment of its generalizability to different language systems such as Chinese, which has a very different ease of mappings between representations. Furthermore, the relative ease of mapping in Chinese is closely linked with the involvement of semantics during lexical processing. Given this, the present paper aimed to investigate the prediction of multiple sources of AoA in Chinese lexical processing.

### AoA effects in Chinese lexical processing

Over 80% of Chinese characters comprise a semantic radical on the left and a phonetic radical on the right (Zhou, 1978). Semantic radicals provide clues to the meanings of the characters, whereas phonetic radicals provide information on how to pronounce the characters. Two measures, *regularity* and *consistency*, are used to quantify the degree to which a phonetic radical provides useful information for pronunciation. Regularity is a measure of whether a character is pronounced the same as its phonetic radical.^1^ Consistency, also known as phonetic radical consistency, is computed by dividing the number of friends (characters sharing the same phonetic radical and pronunciation) by the total number of characters sharing the same phonetic radical (Fang, Horng, & Tzeng, 1986), and the scores can be weighted by character frequency (Lee, Tsai, Su, Tzeng, & Hung, 2005).^2^ The definition of consistency in Chinese parallels the rime (vowel plus final consonant) consistency in English. Compared to English, the print-to-sound mappings in Chinese are much more arbitrary. Therefore, the average consistency score for Chinese characters is much lower than that for English words (0.55 vs. 0.9) (Balota, Cortese, Sergent-Marshall, Spieler, & Yap, 2004; Chang, Welbourne, & Lee, 2016). However, the print-to-meaning mappings in Chinese are relatively regular. Many Chinese characters have semantic radicals that share partial meanings with the characters,^3^ which has proved beneficial for character processing (Dang, Zhang, Wang, & Yang, 2019).

Similar to studies with English words (Monaghan & Ellis, 2002; Cortese & Khanna, 2007; Davies et al., 2017), word naming, which mainly taps phonological processing, and lexical decision, which mainly taps lexical-semantic processing, are prominent paradigms used to investigate AoA effects in Chinese lexical processing (Chen, Zhou, Dunlap, & Perfetti, 2007; Liu, Hao, Shu, Tan, & Weekes, 2008; You, Chen, & Dunlap, 2009). Given that the Chinese writing system is characterized by arbitrary mappings between representations, most studies on AoA reported the findings being consistent with the mapping theory (Chen, Zhou, et al., 2007; Liu et al., 2008; You et al., 2009). For instance, in a character naming task, Liu et al. (2008) demonstrated that AoA effects were stronger for unpredictable characters (irregular and inconsistent) than for predictable characters (regular and consistent). Similar findings were also reported in a lexical decision task (Chen, Dent, You, & Wu, 2009). However, previous mega studies on character naming (Liu, Shu, & Li, 2007) and lexical decision (Sze, Yap, & Rickard Liow, 2015) have demonstrated substantial influence of semantics on both tasks, suggesting that Chinese lexical processing is strongly mediated by semantics. Thus, there is a possibility that the AoA effect could emerge, at least partly, from semantic representations. Although little evidence (Chen, You, & Zhou, 2007; Yum & Law, 2019) has been provided to support the representation theory, Chen, You, and Zhou (2007) demonstrated that the AoA effect was substantially stronger in picture naming than in character naming.

All in all, the current interpretation of AoA in Chinese lexical processing appears to be dominated by the mapping theory. However, semantics processing is also critically involved in both character naming and lexical decision (Liu et al., 2007; Sze et al., 2015). The AoA effect in Chinese could be attributed to multiple sources; however, this possibility has not yet been formally investigated. Thus, the present study was designed to investigate the integrated view of AoA using the large Chinese lexical decision and character naming datasets. The study examined the two key predictions of the integrated view: (a) a reliable AoA effect can be observed in both character naming and lexical decision, and the order of the magnitude of effect sizes in the tasks follows the prediction of the representation theory – the more the semantic involvement in a task, the larger the AoA effect; (b) there is an interaction between AoA and consistency (or regularity) in the character naming task, following the prediction by the mapping theory.

## Method

### Data preparation

Naming data were taken from the psycholinguistic database for traditional Chinese character naming (Chang, Hsu, Tsai, Chen, & Lee, 2016) that includes character naming response times (RTs) for 3,314 characters with, on average, 20 responses per character. The total observations were 66,279, collected from 140 college students in Taiwan. Lexical decision data were taken from normative data of 3,423 traditional Chinese characters (Lee, Hsu, Chang, Chen, & Chao, 2015), with, on average, 30 responses per character. The total observations were 98,733, collected from 180 college students in Taiwan. More details on data collection can be found in the original papers. To compare the psycholinguistic effects across the tasks, only the characters (i.e., 3,314 characters) that were available in both norms were included for analyses.

We tested a range of psycholinguistic predictors on the character-naming and lexical-decision responses. Log character frequency (CF), number of strokes (NS), consistency (CON), regularity (0 for irregular, IREG; 1 for regular, REG; and 2 for unpronounceable phonetic radicals, UNP), phonetic combinability (PC, number of characters that can be created by a phonetic radical), and semantic combinability (SC, number of characters that can be created by a semantic radical) were taken from the psycholinguistic database for character naming (Chang, Hsu, et al., 2016). Semantic ambiguity rating (SAR, measuring the number of meanings of a character) and imageability (IMG, measuring how easily a mental image could be aroused by a character), both based on subjective ratings, were taken from Chang, Hsu, et al. (2016) and Liu et al. (2007), respectively. AoA was computed based on primary-school textbooks of grades 1–6 in Taiwan. All the characters learned after grade 6 were denoted a score of seven. Descriptive statistics of the characters are shown in Table 1.

**Table 1:** Descriptive statistics of the characters included in the analysis

Variable	Mean	SD	Range
Log character frequency (Log CF)	2.02	1.09	0.30–4.78
Number of strokes (NS)	13.18	4.09	4–30
Consistency (CON)	0.55	0.32	0–1
Regularity (IREG, REG, UNP)	0.68	0.66	0, 1, 2
Phonetic combinability (PC)	7.28	4.02	1–20
Semantic combinability (SC)	85.11	66.3	1–226
Age of acquisition (AoA)	5.56	1.95	1–7
Imageability (IMG)	5.07	1.01	2.2–7
Semantic ambiguity rating (SAR)	1.54	0.51	0.92–4.31
Character naming (ms)	741.1	154.84	305–1,116
Lexical decision (ms)	541.2	126.42	301–1,116

### Analysis

We analyzed the data using linear mixed-effect models (LMMs). Following Baayen (2008), an effect was considered to be significant at *p* < .05 level if its t-value was greater than 1.96. In model summaries, we also reported both marginal R^2^ (R^2^_m) and conditional R^2^ (R^2^_c) values; the former is concerned with variance explained by fixed factors while the latter is concerned with variance explained by both fixed and random factors (Nakagawa & Schielzeth, 2013). Prior to analyses, outliers including responses either faster than 300 ms, slower than 2,000 ms, or greater than two standard deviations from the group mean were removed (Table 1). Characters without all the psycholinguistic predictors were also discarded, leaving 22,530 observations for character naming and 38,254 observations for lexical decision analyses.^4^ All the predictors were centered at their means in order to explore interaction terms, and the RTs were z-transformed. The following three analyses were conducted: (a) to investigate the predictability of all the variables, LMM analyses were conducted separately on character naming and lexical decision responses, with item and subject as random factors and all the predictors as fixed factors. To control for potential onset differences, acoustic properties of onset were also included (Balota et al., 2004; Chang, Hsu, et al., 2016; Liu et al., 2007); (b) to investigate the relative involvement of semantics in the character naming and lexical decision tasks, following Davies et al. (2017), a cross-task comparison analysis was conducted by combining the data, and an additional variable, *task,* was created. As the datasets for lexical decision and character naming tasks were collected from different groups of participants, it is likely that the participant characteristics may be different. However, both groups of participants were college students with similar education backgrounds. Moreover, LMM analyses effectively addressed the issue since the random variance due to participant sampling was estimated as a random effect in the models (see Davies et al., 2017, for more discussions). We then investigated each semantic and frequency variable including IMG, SAR, and Log CF, and its interaction with task to see if the pattern of the interaction was similar to that between AoA and task, as predicted by the representation theory (Brysbaert & Ghyselinck, 2006); (c) we investigated if there was an interaction between AoA and consistency, and AoA and regularity in the character naming task, as predicted by the mapping theory (Ellis & Lambon Ralph, 2000).

## Results

The LMM results for character naming and lexical decision analyses are reported in Table 2. For all LMM models, collinearity diagnostic analyses showed variance inflation factors (VIFs) smaller than 3, confirming there was no problem of multicollinearity. For character naming, six out of eight onset features were significant, demonstrating the onset effect in character naming. All the psycholinguistic variables except UNP and PC were significant predictors. For lexical decision, only one onset feature was significant. Log CF, NS, SC, SAR, IMG, and AoA made significant contributions, while REG, UNP, CON, and PC were not significant. The resulting patterns of CON, NS, REG, PC, and SC were largely in line with previous mega studies (Chang, Hsu, et al., 2016; Liu et al., 2007; Sze et al., 2015; Sze, Rickard Liow, & Yap, 2014; Tsang et al., 2018). Of particular interest here are the significant effects of AoA in both character naming and lexical decision tasks. The estimated values for all other lexical-semantic variables including Log CF, SAR, and IMG were numerically larger for character naming (Table 2) than for lexical decision, suggesting that lexical-semantic effects are stronger for character naming than for lexical decision.

**Table 2:** Linear mixed-effect model analyses for character naming and lexical decision

	Character naming			Lexical decision		
	Estimate	SE	T	Estimate	SE	t
*Onset features*						
Stop	-0.028	0.016	-1.731	0.023	0.011	2.165
Aspirated	-0.177	0.019	-9.178	0.006	0.013	0.451
Voiced	-0.083	0.013	-6.185	0.001	0.009	0.088
Bilabial	0.031	0.021	1.479	-0.005	0.014	-0.373
Labiodental	0.099	0.029	3.414	0.005	0.019	0.245
Alveolar	0.068	0.017	4.090	-0.002	0.011	-0.148
Palatoal-veolar	0.156	0.017	9.330	0.007	0.011	0.628
Alveolo-palatal	0.178	0.017	10.325	-0.002	0.011	-0.202
*Variables*						
**Log CF**	**-0.110**	0.007	-15.477	**-0.074**	0.006	-15.795
REG	-0.033	0.013	-2.595	-0.005	0.008	-0.600
UNP	-0.030	0.017	-1.688	-0.009	0.011	-0.745
NS	0.023	0.005	4.183	0.023	0.004	6.158
CON	-0.028	0.007	-4.223	-0.004	0.005	-0.904
PC	0.005	0.006	0.901	-0.006	0.004	-1.684
SC	0.011	0.005	2.177	0.008	0.003	2.224
**SAR**	**-0.034**	0.007	-5.024	**-0.018**	0.004	-3.930
**IMG**	**-0.057**	0.006	-10.371	**-0.030**	0.004	-8.174
**AoA**	**0.030**	0.007	4.515	**0.025**	0.004	5.844
*R*^*2*^*_*m	8.94%	5.63%				
*R*^*2*^*_*c	32.38%	30.18%				

*Log CF* log character frequency, *REG* regular, *UNP* unpronounceable, *NS* number of strokes, *CON* consistency, *PC* phonetic combinability, *SC* semantic combinability, *SAR* semantic ambiguity rating, *IMG* imageability, *AoA* age of acquisition, *R*^*2*^*_m* marginal *R*^*2*^, *R*^*2*^*_c* conditional *R*^*2*^

Four lexical-semantic variables are indicated in bold

### Influence of semantics and AoA on character naming and lexical decision

To statistically examine the influence of semantics and AoA on both tasks, we conducted an LMM analysis, combining character naming and lexical decision data into a dependent variable. This combined model included the variable *task* (0 for character naming and 1 for lexical decision) as an additional predictor together with the fixed and random factors. The combined model was associated with *R*^*2*^*_*m *=* 40.43% and *R*^*2*^*_*c *=* 55.36%, where task was a significant predictor, and *Estimate* = -0.87, *SE* = 0.028, and *t* = -30.88, with faster RTs for lexical decision than for character naming. Furthermore, four interactions were examined using a conservative nested model-comparison approach (Barr, Levy, Scheepers, & Tily, 2013). The significance was assessed by determining whether the model fit improved significantly by applying a likelihood ratio test comparison between models with and without the interaction of interest. Task by Log CF, SAR, IMG, or AoA was added into the combined model separately as a fixed factor. Adding Log CF × task to the model resulted in a significant improvement, χ^2^(1) = 87.97, *p <* .001, where *R*^*2*^*_*m and *R*^*2*^*_*c increased to 40.49% and 55.45%, respectively. Adding SAR × task resulted in a significant improvement, χ^2^(1) = 73.98, *p <* .001, where *R*^*2*^*_*m and *R*^*2*^*_*c increased to 40.48% and 55.43%, respectively. Adding IMG × task also resulted in a significant improvement, χ^2^(1) = 65.22, *p <* .001, where *R*^*2*^*_*m and *R*^*2*^*_*c increased to 40.48% and 55.42%, respectively. A similar result was also observed for AoA × task, χ^2^(1) = 114.72, *p <* .001, where *R*^*2*^*_*m and *R*^*2*^*_*c increased to 40.51% and 55.47%, respectively.

**Fig. 1:**
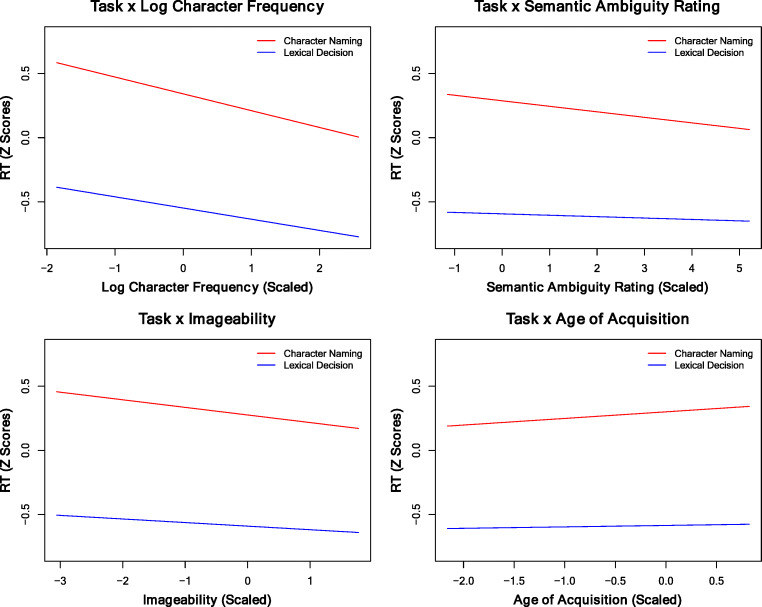
The interaction patterns between task and log character frequency, semantic ambiguity rating, imageability, and age of acquisition, respectively

The interaction patterns (Fig. 1) showed that all target effects were stronger for character naming than for lexical decision. Note that although the RTs were longer for character naming than for lexical decision, not all the psycholinguistic effects were stronger for character naming (see additional comparisons^5^).

### AoA and consistency/regularity interaction in character naming

Lastly, we examined whether there was an interaction between AoA and consistency or regularity in the character naming task. The LMM analysis of character naming (Table 2) was used as the baseline model. Adding AoA × consistency resulted in a significant improvement, χ^2^(1) = 4.38, *p =* .036, *R*^*2*^*_*m *=* 8.95%, and *R*^*2*^*_*c *=* 32.37%. Adding AoA × regularity did not result in any improvement, χ^2^(2) = 0.62, *p =* .73, though it was found to be significant when the three-way interaction (frequency × AoA × regularity) was added, χ^2^(3) = 26.21, *p <* .001, *R*^*2*^*_*m *=* 9.01% and *R*^*2*^*_*c *=* 32.33%. Thus, the interaction between AoA and regularity seems to be crossed with frequency.

## Discussion

This study aimed to examine the AoA effects in Chinese character naming and lexical decision based on a large sample of items and participants. The LMM analyses showed that AoA accounted for unique variance in character naming and lexical decision. By comparing the effect size of frequency and semantic variables including IMG and SAR in character naming and lexical decision, it was found that the influence of semantics was stronger for character naming than for lexical decision, which is different from the patterns observed in English language (Brysbaert & Ghyselinck, 2006; Chang et al., 2019; Cortese & Khanna, 2007). However, the magnitude of AoA effect was in line with that of semantic and frequency effects, and the AoA effect was also stronger for character naming than lexical decision. The results suggest that AoA has a common origin with lexical-semantic variables, as predicted by the semantic representation theory (Brysbaert & Ghyselinck, 2006). The additional comparison demonstrated a significant interaction between AoA and consistency (or regularity with moderation by frequency) in character naming, thus supporting the mapping theory. Overall, the findings of AoA in Chinese show interesting divergent patterns from that in English. Importantly, though, the patterns follow the predictions of the integrated view of multiple sources of AoA (Brysbaert & Ellis, 2016; Chang et al., 2019; Dirix & Duyck, 2017; Menenti & Burani, 2007).

Different theories have been proposed to explain the origin of AoA (see Brysbaert & Ellis, 2016). The mapping theory proposes that the AoA effect is due to reduced neuroplasticity when more words are learned (Ellis & Lambon Ralph, 2000). The AoA effects are expected in an arbitrary mapping between representations (Zevin & Seidenberg, 2002). Alternatively, AoA effects can be associated with semantic representations, where early-learned words tend to have richer representations and more connections with other words (Brysbaert et al., 2000). When semantics is required, the AoA effects are observable and the magnitude depends on semantic involvement. A recent integrated view of AoA, however, suggests that both the development of representations and the mappings between representations could influence lexical-semantic processing, contributing to AoA effects (Brysbaert & Ellis, 2016; Chang et al., 2019; Dirix & Duyck, 2017; Menenti & Burani, 2007). Given that Chinese has a deep orthography system, the print-to-sound mappings are largely arbitrary. A strong AoA effect and its interaction with consistency, predicted by the mapping theory, has been shown in several studies of AoA in Chinese character naming (Chen, Zhou, et al., 2007; Liu et al., 2008; You et al., 2009). Similar interactions are also found in the present study. However, when the differential AoA effects in character naming and lexical decision tasks were directly investigated in the present study, the magnitude of the AoA effect was found to be linked with semantic involvement, as predicted by the representation theory. The findings collectively suggest that AoA cannot be solely determined by the arbitrariness of mappings but is also related to the construction of representations. Accordingly, AoA effects are more likely to stem from multiple sources, suggesting that learning experience could affect the processing in terms of the development of connections within representations as well as the mappings between representations across languages.

The cross-task comparison approach used in this study is theoretically important for the explanation of AoA effects, allowing for the concurrent investigation of predictions from alternative theories. The results of the cross-task comparison established the relative influence of frequency and semantics in Chinese character naming and lexical decisions, the effect of the former being greater than that of the latter. This directionality of the effect diverges from the findings in English, where semantics is more involved in lexical decision than in word naming (Brysbaert & Ellis, 2016; Brysbaert & Ghyselinck, 2006; Chang et al., 2019; Cortese & Khanna, 2007). This is likely because more than half of the Chinese characters do not have systematic print-to-sound mappings (Chang, Hsu, et al., 2016). The access to semantics for character naming is vital, as has been stated by several mega studies (Chang, Hsu, et al., 2016; Chang & Lee, 2018; Liu et al., 2007; Sze et al., 2014; Sze et al., 2015) and computational-modeling studies (Chang, Welbourne, & Lee, 2016). It is worth noting that the effect of the cross-task comparison (indexed by interaction) in the present study was small albeit reliable. Large differences could be anticipated when comparing tasks closely linked to semantic processing (e.g., picture naming) relative to lexical-semantic tasks. As demonstrated by Chen, You, and Zhou (2007) and our additional comparisons (see Online Supplementary Material), the AoA effect is much stronger for picture naming than for character naming, followed by lexical decision. However, both investigations are based on small sample sizes. A large-scale and systematic comparison across multiple tasks could be a key topic for future investigation. One potential limitation of this study is that the cross-task comparison was based on different samples of participants. However, the issue of participant sampling was overcome as the participants had a similar educational level and individual differences were estimated as random variance during the analyses. Future cross-task comparisons can be conducted using a within-subject design for further improvement.

In conclusion, this study investigated the AoA effects by conducting a cross-task comparison between Chinese character naming and lexical decision. Even though Chinese lexical processing is characterized by different ease of mappings compared to English, the impact of individual learning experience is general, affecting both the incremental construction of representations and the learning of mappings between representations.

## Electronic supplementary material

ESM 1 (DOCX 121 kb)

## Data Availability

Both the Chinese character naming and lexical decision data used in the current study can be downloaded from the Traditional Chinese Psycholinguistic Database website (http://ball.ling.sinica.edu.tw/namingdatabase/AoA.html).

## Footnotes

1 For instance, 清 (/ching1/) is a regular character because its pronunciation is the same as its phonetic radical 青 (/ching1/), while 灑 (/sa2/) is an irregular character because its pronunciation is different from its phonetic radical 麗 (/li4/).

2 For instance, there are eight characters sharing the same phonetic radical 登 (/deng1/), but only 橙 and 澄 are pronounced in the same way (/cheng2/). Thus, 橙 and 澄 have a consistency value of 0.25, and the frequency-weighted consistency value would be 0.243.

3 For instance, 媽 (/ma1/) means *mother* and its semantic radical 女 (/niu3/) means *female*.

4 The loss of data observations was primarily due to the substantial lack of IMG ratings for the characters used in the study. We also conducted the same LMM analyses after removing IMG, with 46,980 observations for character naming and 85,402 observations for lexical decision. The results showed that the key patterns of AoA remained the same. In particular, with the removal of IMG, the estimate value for AoA increased from 0.03 to 0.07 for character naming (*t* = 13.72), and from 0.025 to 0.04 for lexical decision (*t* = 11.79). These analyses demonstrated that data exclusion did not bias the results; rather, there was shared variance between IMG and AoA. Therefore, we included IMG in our analyses.

5 For comparison, the interactions between NS × task and SC × task were examined. The results showed that the interaction between NS × task was not significant, *p =* .74, while the SC effect was stronger for lexical decision than for character naming, χ^2^(1) = 5.36, *p =* .021, *R*^*2*^*_*m *=* 40.43% and *R*^*2*^*_*c *=* 55.37%.
