# Supplementary material for: Age of acquisition effects on traditional Chinese character naming and lexical decision
Source: Psychon Bull Rev. 2020 Aug 12;27(6):1317–24. doi: 10.3758/s13423-020-01787-8 (PMC7704508; doi:10.3758/s13423-020-01787-8)
Supplement: Supplementary file 1 — (DOCX 121 kb) [file 13423_2020_1787_MOESM1_ESM.docx]

**Supplementary**

**AoA in Picture Naming, Character Naming and Lexical Decision**

We compared the AoA effect in picture naming, character naming and lexical decision. The picture naming data were taken from Liu, Hao, Li, and Shu (2011), and the character naming and lexical decision data were from the present study. In Liu et al. (2011), the norm contained 435 objects; however, most of them have polysyllabic names, and only average response times (RTs) are available. Thus, we overlapped the item names between their objects and our words, leaving 28 items. We then conducted regression analyses. The RTs were used as a dependent variable. The predictors included Log frequency (LogFreq) and age of acquisition (AoA), taken from Liu et al. (2011). Task with three levels, lexical decision (Task_LD), character naming (Task_CN), or picture naming (Task_PN), was also included as a predictor, in which Task_CN was used as a reference level. Both LogFreq and AoA were centred at their means and the RTs were z-transformed. The result showed the regression model was associated with adjusted *R*^2^ = 48.71%. AoA was a significant predictor, *Estimate* = 0.23, *p* = .011, while LogFreq did not reach significance (*p* = 0.37). Task_LD was a significant predictor, *Estimate* = -0.88, *p* < .001, indicating relative to character naming, RTs for lexical decision were significantly shorter. Whereas, Task_PN was not significant, *p* = 0.28, indicating no difference between character naming and picture naming. Adding an interaction between task and AoA resulted in *R*^2^ increased to 64.72%, in which the AoA effect was stronger for picture naming than character naming and then lexical decision, as in Figure S1. The resulting pattern of AoA provided additional support to the integrated view of AoA illustrated in the main text. The null effect of frequency was similar to the findings of Liu et al. (2011) and Weekes et al. (2007) on picture naming. It is likely because the sample size was small so that the effect was not observable when AoA was controlled.

Figure S1. The interaction pattern between task (Character naming, Lexical decision and Picture naming) and AoA.

**Supplementary References**

Liu, Y., Hao, M., Li, P., & Shu, H. (2011). Timed Picture Naming Norms for Mandarin Chinese. *PLOS ONE, 6*(1), e16505. doi: 10.1371/journal.pone.0016505

Weekes, B. S., Shu, H., Hao, M., Liu, Y., & Tan, L. H. (2007). Predictors of timed picture naming in Chinese. *Behavior Research Methods, 39*(2), 335-342. doi: 10.3758/BF03193165
